# Prediction of crossover recombination using parental genomes

**DOI:** 10.1371/journal.pone.0281804

**Published:** 2023-02-16

**Authors:** Mauricio Peñuela, Camila Riccio-Rengifo, Jorge Finke, Camilo Rocha, Anestis Gkanogiannis, Rod A. Wing, Mathias Lorieux

**Affiliations:** 1 Facultad de Ingeniería y Ciencias, Pontificia Universidad Javeriana, Cali, Colombia; 2 AgroBiotechnology Unit, Alliance Bioversity-CIAT, Cali, Colombia; 3 Arizona Genomics Institute, University of Arizona, Tucson, AZ, United States of America; 4 DIADE, University of Montpellier, CIRAD, IRD, Montpellier, France; University of Guelph, CANADA

## Abstract

Meiotic recombination is a crucial cellular process, being one of the major drivers of evolution and adaptation of species. In plant breeding, crossing is used to introduce genetic variation among individuals and populations. While different approaches to predict recombination rates for different species have been developed, they fail to estimate the outcome of crossings between two specific accessions. This paper builds on the hypothesis that chromosomal recombination correlates positively to a measure of sequence identity. It presents a model that uses sequence identity, combined with other features derived from a genome alignment (including the number of variants, inversions, absent bases, and CentO sequences) to predict local chromosomal recombination in rice. Model performance is validated in an inter-subspecific *indica* x *japonica* cross, using 212 recombinant inbred lines. Across chromosomes, an average correlation of about 0.8 between experimental and prediction rates is achieved. The proposed model, a characterization of the variation of the recombination rates along the chromosomes, can enable breeding programs to increase the chances of creating novel allele combinations and, more generally, to introduce new varieties with a collection of desirable traits. It can be part of a modern panel of tools that breeders can use to reduce costs and execution times of crossing experiments.

## Introduction

Crossover recombination refers to the exchange of genetic material across homologous chromosomes. It is an important process during meiosis in the production of gametes and contributes to the creation of novel allele combinations [1–3]. Both biological and biochemical factors influence the recombination rates along each chromosome. In rice, for example, it has been shown that recombination rates play a key role for adaptive evolution in rapidly changing environments and vary with exposure to different stresses [4]. Furthermore, a number of studies have shown that recombination rates across different regions along a chromosome (i.e., for windows of a certain size) are not uniformly distributed [5, 6]. Instead, there exists the so-called hot and cold spots, which represent regions that, when compared to regular regions, exhibit relatively high and low rates of recombination. According to [4, 7, 8], the location of such regions varies between populations, primarily as a result of population history.

Over generations, recombination has played an important role in the evolution of the genome in plants [6]. Evidence suggests that recombination responds not only to direct selection, but also to the effects of indirect selection over different traits [7]. From the perspective of agricultural growth and development, understanding recombination rates enables plant breeders to develop better criteria for determining: (i) which varieties represent the most suitable parents for crosses and (ii) which progeny make the selection process highly effective [9]. More specifically, estimating the recombination rates along the chromosomes accelerates the fine mapping of genetic traits [10], which lies at the heart of efforts to design better crops [2].

The design and development of experiments to measure recombination rates between varieties is a demanding task, both in terms of costs and time. Such efforts require, first, a large number of recombinant descendants and, second, a large number of markers from high throughput next generation sequencing. Not surprisingly, several studies have introduced different strategies to characterize recombination rates in the chromosomal arms [2, 3, 8, 11–15]. These studies generally evaluate several varieties to construct a genomic recombination landscape for a species as a whole. They tend to follow one of two general approaches. One main approach seeks to discover and understand which factors explain recombination, identifying features of the genome, and searching for associations with high or low levels of recombination. The second main approach aims to predict either the location of hot and cold spot, or to estimate the recombination rates in the chromosome using different types of genome sequence information by usually applying machine learning models.

Following the first approach, the work by Rodgers-Melnick et al. [11] identifies recombination breakpoints in populations of U.S. and Chinese maize. The authors show that the distribution of gene density and CpG methylation explains, on a broad scale, cross-overs. In another closely-related study, Colomé-Tatché et al. [12] evaluate the combined effect of removing sequence polymorphisms and repeat-associated DNA methylation on the meiotic recombination landscape of an Arabidopsis mapping population. Similarly, Horton et al. [13] test 1, 307 worldwide Arabidopsis accessions to characterize the pattern of recombination history. The authors observe an enrichment of hot spots in regions of intergenic space and repetitive DNA. Finally, Haas et al. [2] identify AT-rich DNA motifs associated with recombination breakpoints in 60 recombinant inbred lines of tomato.

One of the first studies to follow the second approach is the work by Liu et al. [8]. Based on sequence *k*-mer frequencies, the authors predict hot and cold spots in yeast using a machine learning method known as increment of diversity combined with quadratic discriminant analysis. The work is extended in [14] by introducing an algorithm to predict hot and cold spots in yeast. Unlike [14], the work by Demirci et al. [15] applies features related to genome content and genomic accessibility, such as gene annotation, propeller twist and helical twist, and AT/TA dinucleotides to train different machine learning models (specifically, decision trees, logistic regression, and random forest models). Their work predicts hot and cold spots in maize, rice, tomato, and Arabidopsis. A more recent work by Adrion et al. [3] proposes a method to predict the recombination landscape based on deep learning algorithms; they evaluate model predictions in African populations of *Drosophila melanogaster*. Finally, Peñuela et al. [16] trained an extra trees machine learning model to predict recombination in rice using methylated cytosines in the CHH context.

A number of studies that follow the second approach characterize broad-scale recombination rates for windows of certain size along a chromosome. They tend to focus on a given population or species. However, little attention has been paid to developing analytical frameworks that help explain recombination rates for a specific crossing between two particular varieties. The lack of such models limits the applicability of the outcome of studies that follow the second approach for breeding programmes. To overcome this limitation, the validation of such models is required. The lofty aim of the mechanism-based models is that the principles for prediction are generalizable and applicable to other varieties or species.

This paper focuses on predicting specific recombination rates that result as the product of a crossing between the rice (*Oryza sativa* L.) varieties of IR64 (indica) and Azucena (japonica). Since they are genetically distant varieties, developing a prediction of recombination between them may shed light on predicting recombination in closer varieties. A large number of studies that aim to estimate recombination rates focus on rice for several reasons. Among them, rice is highly homozygous, which makes haplotype reconstruction easy and also eliminates the need of phasing. Moreover, rice provides food for more than half the world’s population [17]. In particular, this work explores the hypothesis that an identity measure between genome sequences of the parents is correlated with chromosomal recombination. The analysis is performed based on whole genome sequencing of both rice varieties and their recombinant inbred lines.

The main result of this work suggests that the sequence identity is positively correlated with chromosomal recombination. The model can predict recombination using parental sequences as its input. Unlike the above-mentioned models based on machine or deep learning approaches, this is a mechanism-based model whose outcome is the result of a series of steps applied to specific features measured after the alignment process between parental sequences. The model is calibrated on Chromosome 1 and tested on the remaining 11 chromosomes. The validation of the model shows that the prediction for the rice chromosomes has an average correlation of 80% with the recombination rates. It has the potential to become a tool improving plant breeding programs in rice cultivars.

## Materials and methods

The IR64 (indica cluster) and Azucena (tropical japonical cluster) varieties were crossed to generate a F1 generation. A total of 212 F8 recombinant inbred lines (RIL) were generated in the greenhouse at IRD, France, by single-seed descent (SSD) from the F2. Then, the lines were advanced in the field to the F12 generation at the International Center for Tropical Agriculture (CIAT, now “Alliance Bioversity-CIAT”) in Palmira, Colombia. This population is also part of a Nested association Mapping design [18].

### Whole genome sequencing

Leaf tissue from parent plants and F12 lines were collected, and DNA was extracted following a protocol similar to [18]. Platinum-grade PacBio assemblies of the parental genomes were obtained at the Arizona Genomics Institute (AGI, Tucson, Arizona) [19]. The IR64 and Azucena genomes that were used are available in the GenBank repository with the accession numbers RWKJ00000000 and PKQC000000000, respectively. The F12 RIL genomes were sequenced using paired-end Illumina with a depth of approximately 1x.

### Data imputation and recombination values

SNP features for the F12 genomes were extracted using a standard bioinformatics pipeline. Briefly, Illumina reads were mapped on the IR64 RefSeq, and SNP features were extracted with the GATK package. Genotypes and recombination breakpoints (that is, meiotic crossovers) were imputed and corrected using the NOISYmputer algorithm introduced in [20]. The resulting genotypes data for each chromosome consist of a matrix of genetic markers (arranged by sequence position) versus individuals. An entry is encoded as A or B depending on the parental origin of the corresponding sequence. Genetic recombination maps were calculated with MapDisto v2 [21, 22], using the Kosambi mapping function to convert recombination fractions into centimorgans (cM) [23].

### Recombination measurement

Cublic spline smoothing of local recombination rates, expressed as cM/bp, were calculated in sliding windows in MapDisto v2. A window size of 100 kb was chosen to measure recombination because it provides a detailed description of how crossovers occur along the chromosome. Especially, it helps to find out what exactly happens in regions where recombination rates are high. When the window size is larger, like 1 Mb for example, the recombination rates of the windows can be very high due to the accumulation of many crossover events. The problem is that it is not possible to know where these crossovers are located, they can all be at the beginning of the window, or at the end, and they can even be evenly distributed throughout the window. Large window sizes can also lead to more noise in the data, because neighboring windows can vary widely, making them difficult to handle in statistical analyzes. In addition, by increasing the window size, the number of windows per chromosome decreases, which makes it difficult to train the models to make and evaluate predictions. On the other hand, if the window size is smaller, few crossover events can be count for window, it would be necessary to have a larger experiment with a much larger number of RILs to be able to obtain counts for most windows. Experiments were developed to find an appropriate window size for our data and objectives; according to them, the 100 kb window size was chosen because it results in a significant number of crossover events without losing precision.

### Data pre-processing protocol

The purpose of this work is to predict recombination for each pair of homologous chromosomes from two parental organisms. The proposed approach is based on the hypothesis that the recombination frequency can be approximated by a function of the genome similarity. To measure genome similarity, a metric called *identity* was constructed taking into account features of the alignment of the two parental sequences.

Arbitrarily, one of the parental organisms is taken as reference. Each pair of homologous chromosomes is identified by a reference chromosome (*ref*) and a query chromosome (*qry*). Each pair (*ref*, *qry*) is aligned using the MUMmer3 [24] software. The *nucmer* command with default parameters performs the initial alignment. The outcome is a delta file which is filtered using the command delta-filter -r -q. The filtered file is used to extract coordinates into a coords file, using the command show-coords -r. Sequence variants are extracted into a snps file, from the initial delta file using the command show-snps.

Subsequently, and using Python software from this point on, the reference chromosome sequence is subdivided into n∈N>0 windows of length 100 kb each. Three features for each window are computed from the coords and snps files:

- Inversions: proportion of reference bases belonging to regions aligned in the reverse direction (3’-5’).
- Absent bases: proportion of query bases that are not mapped in the reference chromosome.
- Variants: proportion of bases corresponding to SNPs and deletion polymorphisms.

The identity criteria is concretely defined in terms of the three above-mentioned features. It is also parametric on the windows partitioning a chromosome. Let *W* = {1, 2, …, *n*} represent the *n* windows partitioning a given chromosome. Functions *I*, *A*, and *V* next represent the inversions, absent bases, and variants measures, respectively. They are defined from the set *W* of windows to the closed real interval [0, 1]. More specifically, these functions are defined as *I*: *W* → [0, 1], *A*: *W* → [0, 1], and *V*: *W* → [0, 1]. The identity criteria function *Id*_0_ maps the set of windows to a real number: the higher its value, the closer the two sequences genetically are in the given window. In other words, the identity is equal to 1 if the two compared windows are identical, but in the presence of inversions, absent bases, or variants, the identity is decreased. Mathematically, *Id*_0_ is defined for each window *w* ∈ *W* by the equation:

$$\begin{array}{c} Id_{0}\left(w\right)=1-\left(V\left(w\right)+I\left(w\right)+A\left(w\right)\right). \end{array}$$ (1)

For each window *w*, *Id*_0_(*w*) quantifies a genetic distance between two (parental) sequences where variants, inversions, and absent bases are used to linearly penalize the identity measure. This criteria is used for pre-processing each pair of parental sequences and it is at the basis of the proposed model for recombination prediction.

### Testing hypothesis

**Fig 1:**
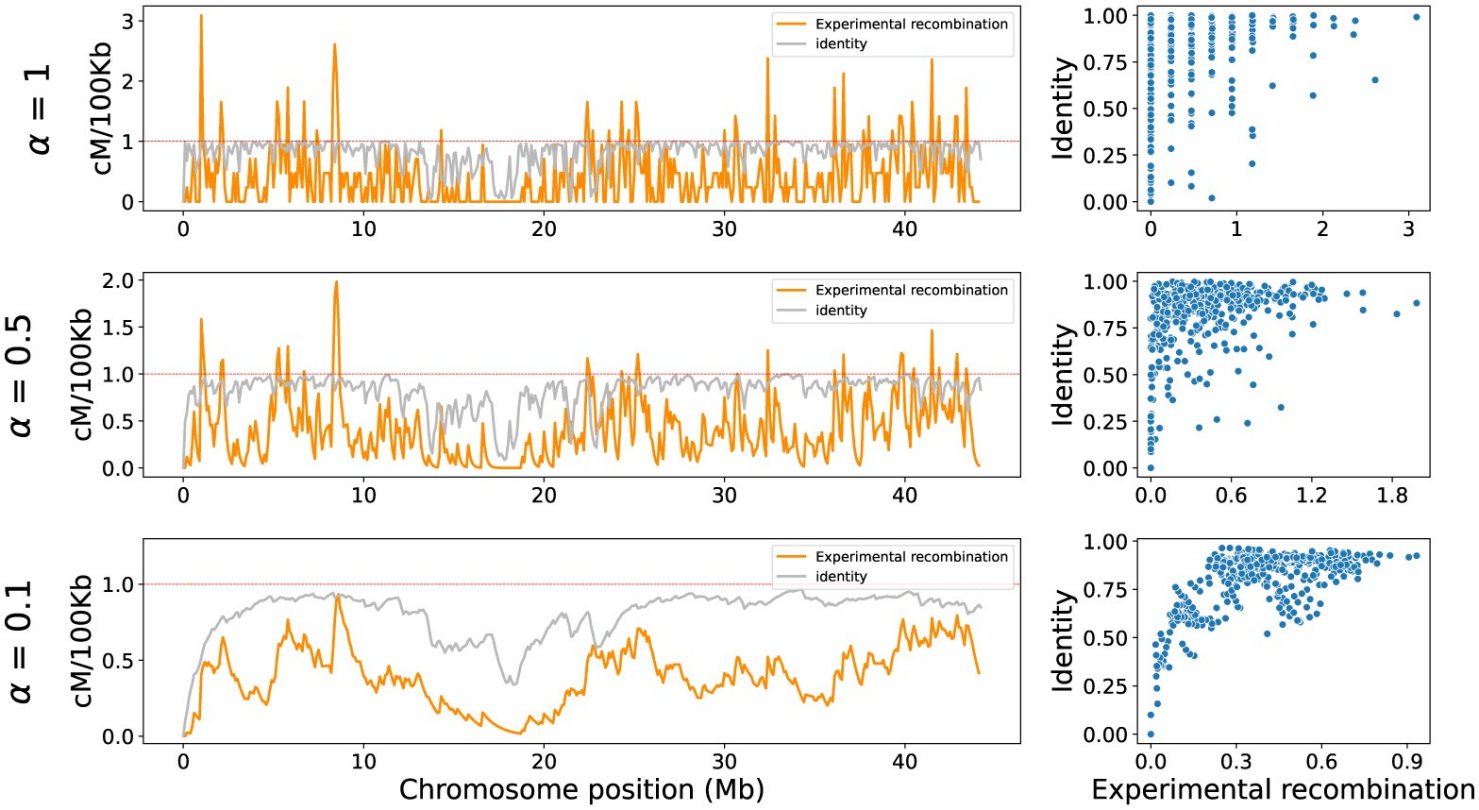
Effect of exponential smoothing on recombination and identity signals along the chromosome. The graphs on the left show the chromosome recombination rate in orange and identity values in grey at different levels of smoothing, where *α* = 1 is no smoothing, *α* = 0.5 an intermediate smoothing, and *α* = 0.1 a strong smoothing (where the noise disappears). The horizontal red line is a reference that helps to visualize the decrease of large recombination values. The scatter plots on the right show the relationship between identity and experimental recombination at each smoothing level; the dots represent the 100 kb windows of the left graphs.

**Fig 2:**
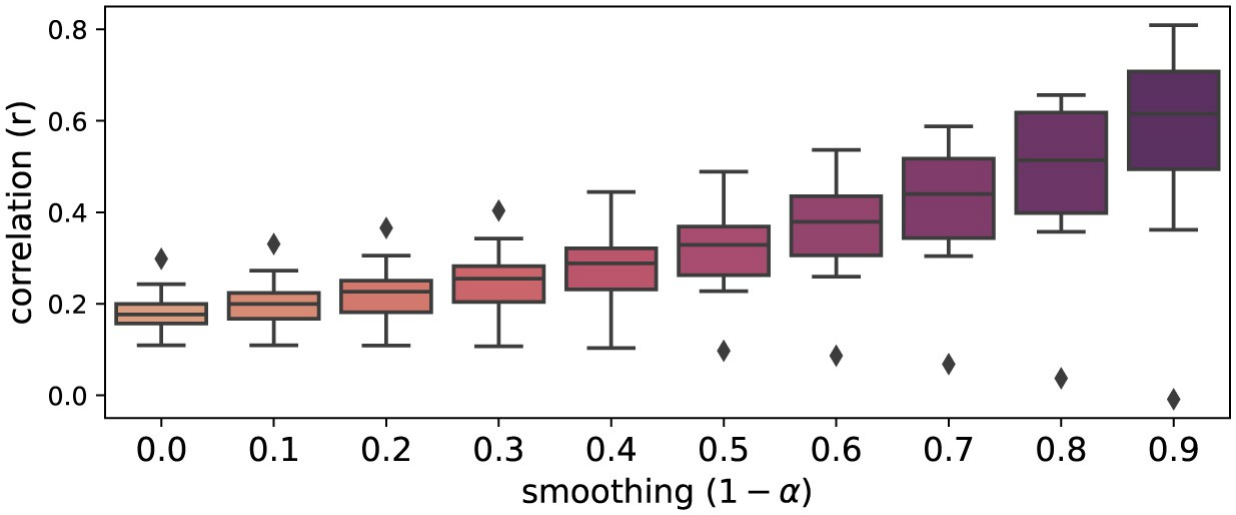
Effect of exponential smoothing on correlation distribution. Boxplots of correlation between identity and recombination for 12 rice chromosomes (cross IR64 x Azucena) at different levels of exponential smoothing. Note that identity is a ratio while experimental recombination is a rate in cM/100kb. The correlation values increases as the smoothing value increase, thus reducing noise.

Under the hypothesis that similar genomic regions recombine more frequently, a correlation analysis was developed between the identity criteria and the local recombination values for the twelve rice chromosomes. The Pearson’s correlation coefficient was used as the measure of correlation *r*. The identity and the recombination were exponentially smoothed to reduce noise and find the best fit with the trend of the data. For example, functions *X* and *X*_*s*_ represent the experimental recombination and the smoothed experimental recombination, respectively. Both functions are defined for each window *w* ∈ *W*; in particular, *X*_*s*_ is defined by the equation:

$$\begin{array}{c} X_{s}\left(w\right)=\{\begin{array}{cc} X(w) & w=0\\ \alpha X\left(w\right)+\left(1-\alpha \right)X_{s}\left(w-1\right) & w>0, \end{array} \end{array}$$ (2)

where *α* ∈ (0, 1) is the smoothing factor. For the correlation analysis, both identity and experimental recombination were smoothed with the same factor. Various exponential smoothing factors were evaluated in each chromosome to try to reduce noise and find the best fit with the data trend (Figs 1 and 2), being *α* = 0.1 the one giving the best fit in all cases. This smoothing factor was selected and applied to subsequent evaluations on the model predictions.

### Model

**Fig 3:**
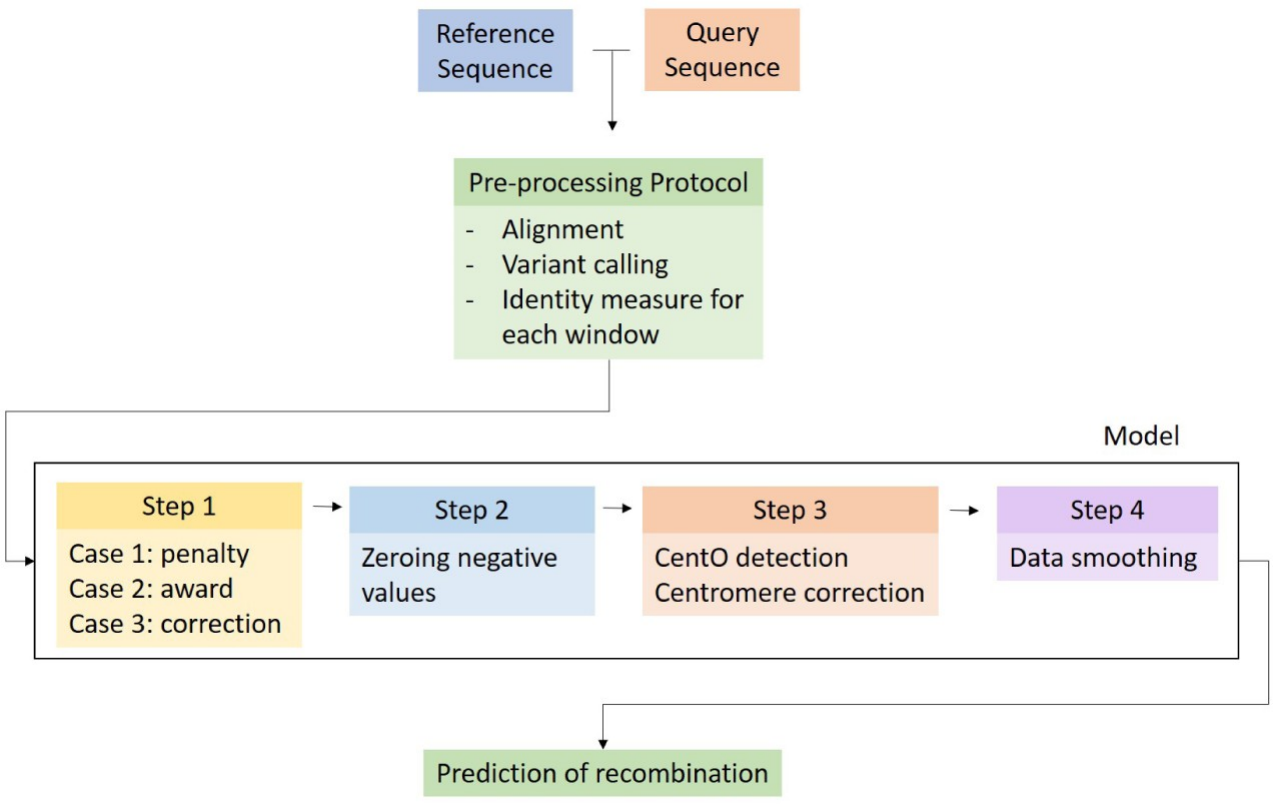
Model workflow. Schematic representation of data preprocessing and model steps to predict recombination. The preprocessing protocol receives two parental sequences as input and produces a measure of identity between the two sequences. The model receives this identity as input and outputs the predicted chromosomal recombination rate.

A four-step model based on alignment data is developed. The first step applies three cases to modify the identity of each window to maximize the effect of zones with low and high identity values. The second step adjusts the output so that negative values with no biological interpretation are corrected. The third step performs a centromeric correction based on CentO sequences to improve the prediction of low recombination near the centromere. Finally, the fourth step implements a smoothing to reduce noise, allowing a cleaner evaluation of the predictions. The model contains 7 parameters which transform the identity to predict the recombination rate by each window (Fig 3). A model implementation in Python is publicly available at https://github.com/criccio35/Rice-recombination-predictor.

#### Step 1: Cases

In the first step of the model, three cases are defined to alter the identity of some windows, and to better fit valleys and peaks of real recombination using sequence information.

The first case, the penalty stage, is coherent with the idea that regions with low identity recombine less. Therefore, a window with low identity value should be penalized (further decrease its value), in contrast to a window with high identity values that should remain intact. More precisely, a constant value is subtracted from the windows where the non-identical part has a considerable influence of the variants. This stage causes regions with such features to form valleys, thus increasing the correlation with chromosomal recombination rates. Biologically, these adjustments model the fact that few recombination events are expected if there is no high genomic identity between parental chromosomal regions. This observation is in accordance with the initial hypothesis of this study.

The second case, the reward stage, consists of rescuing windows with low identity values and small influence from the variants. The reason for doing this is that there could be alignment fragments with high (almost perfect) identity values, and with size smaller than the 100 Kb window and having low variants proportion. Therefore, this case is useful to predict recombination peaks in regions with low or average identity.

The third case, the correction stage, is included in order to deal with windows with an over-adjustment in the alignment process; mainly, windows with high identity values that are not dealt with by the previous two cases. Specifically, the correction consists of subtracting a constant factor from the identity values with absent bases and low influence of the variants. If there are absent bases in a window, it means that the data in the window is constructed from more than one contig. Furthermore, such a window contains few variants, probably because the information depends on multiple contigs that do not accurately represent the structure of the corresponding chromosomal region. For windows in which none of the three previous cases are applied, the initial identity values are assigned.

Mathematically speaking, summarizing the cases explained above, three mutually exclusive cases are considered starting from the identity values mapped by the function *Id*_0_. The model has a total of 7 parameters which belong to the closed interval [0, 1]. The parameters are classified into two groups: (i) the constant factors *p*_1_, *p*_2_, and *p*_3_ that modify the identity values in each case, and (ii) the thresholds *t*_1_, *t*_2_, *t*_3_, and *t*_4_ that define when to apply the cases. The first case penalizes with *p*_1_ the identity of those windows with identity values inferior to *t*_1_. The second case rewards with *p*_2_ the windows with identity values inferior to *t*_2_. The third case penalizes with *p*_3_ the windows with absent bases greather than *t*_3_. An additional constraint to apply case one is that the variants must be above *t*_4_, while for the cases two and three variants must be below the same threshold (*t*_4_). Thus, identity values are updated with the function *Id*_1_ defined from the set *W* of windows to the closed real interval [−1, 2], that is *Id*_1_: *W* → [−1, 2]. For each window *w* ∈ *W*, *Id*_1_ is defined as:

$$\begin{array}{c} Id_{1}\left(w\right)=\{\begin{array}{ccc} Id_{0}\left(w\right)-p_{1} & , & Id_{0}\left(w\right)<t_{1}\wedge V\left(w\right)>t_{4}\\ Id_{0}\left(w\right)+p_{2} & , & Id_{0}\left(w\right)<t_{2}\wedge V\left(w\right)<t_{4}\\ Id_{0}\left(w\right)-p_{3} & , & A\left(w\right)>t_{3}\wedge V\left(w\right)<t_{4}\\ Id_{0}\left(w\right) & , & \text{otherwise.} \end{array} \end{array}$$ (3)

**Fig 4:**
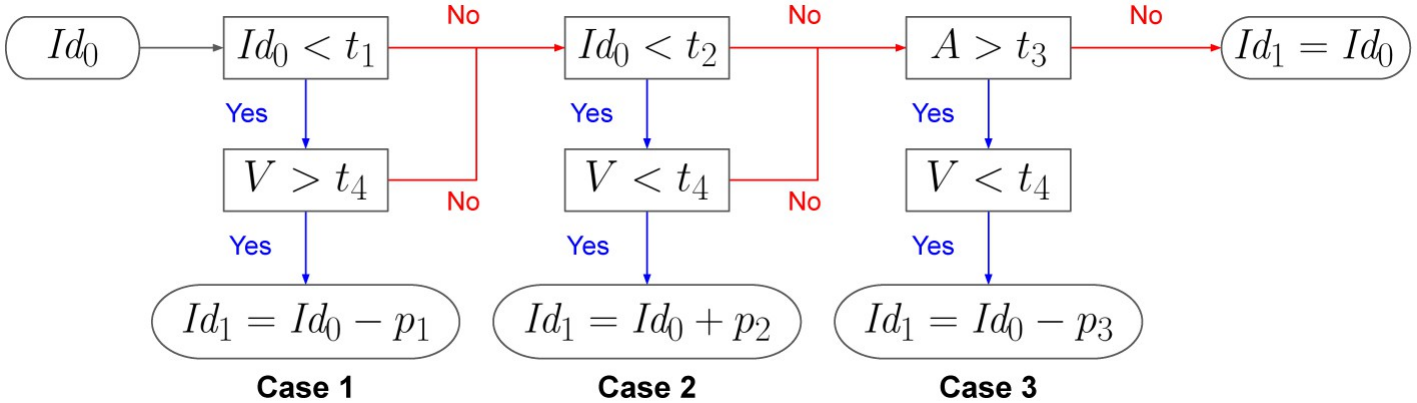
Decision tree to modify the identity value in Step 1. The window identity can be modified according to different thresholds *t* for; the identity values *Id*_0_, the variants *V* and the absent bases *A*. In each case a modifcation is applied to the identity value adding or subtracting a constant factor *p*. If no case is applied, the identity value remains unchanged.

Fig 4 presents a graphical description of the identity modification process, as a decision diagram, according to the thresholds that are validated in each case.

#### Step 2: Negative values

The second step of the model consists of zeroing the negative values resulting from the first step. This is necessary because, biologically, recombination rates are always positive and negative recombination values do not make biological sense. Therefore, only non-negative values are considered. Mathematically, this step produces a function *Id*_2_ defined from the set of windows *W* to the real closed interval [0, 2]. More specifically, the new updated identity *Id*_2_: *W* → [0, 2] is defined for each *w* ∈ *W* as:

$$\begin{array}{c} Id_{2}\left(w\right)=\text{max}\left(0,Id_{1}\left(w\right)\right) \end{array}$$ (4)

#### Step 3: Centromere correction

The third step of the model attempts to predict the boundaries of the centromeric region and adjust the nearby identity values. CentO(AA) sequence reported by Lee et al. [25] is mapped on the reference and query chromosomes counting the frequency of aligned bases within each window. Let *wcentO* be a function that maps a chromosome to the set of windows having the greatest number of alignments with the CentO sequence. Note that *wcentO* outputs a non-empty subset of the set of windows *W* for both reference (*ref*) and query (*qry*) chromosomes (*wcentO*(*ref*) ∪ *wcentO*(*qry*) ⊆ *W*). Then, the centromere boundaries can be approximated by the interval [*c*_0_, *c*_1_] defined by:

$$\begin{array}{c} c_{0}=\text{min}\left(wcentO\left(ref\right)\cup wcentO\left(qry\right)\right) \end{array}$$ (5)

$$\begin{array}{c} c_{1}=\text{max}\left(wcentO\left(ref\right)\cup wcentO\left(qry\right)\right) \end{array}$$ (6)

That is, *c*_0_ and *c*_1_ are the left- and right-most windows with the greatest number of alignments with the CentO sequence, among the two chromosomes input to the model.

**Fig 5:**
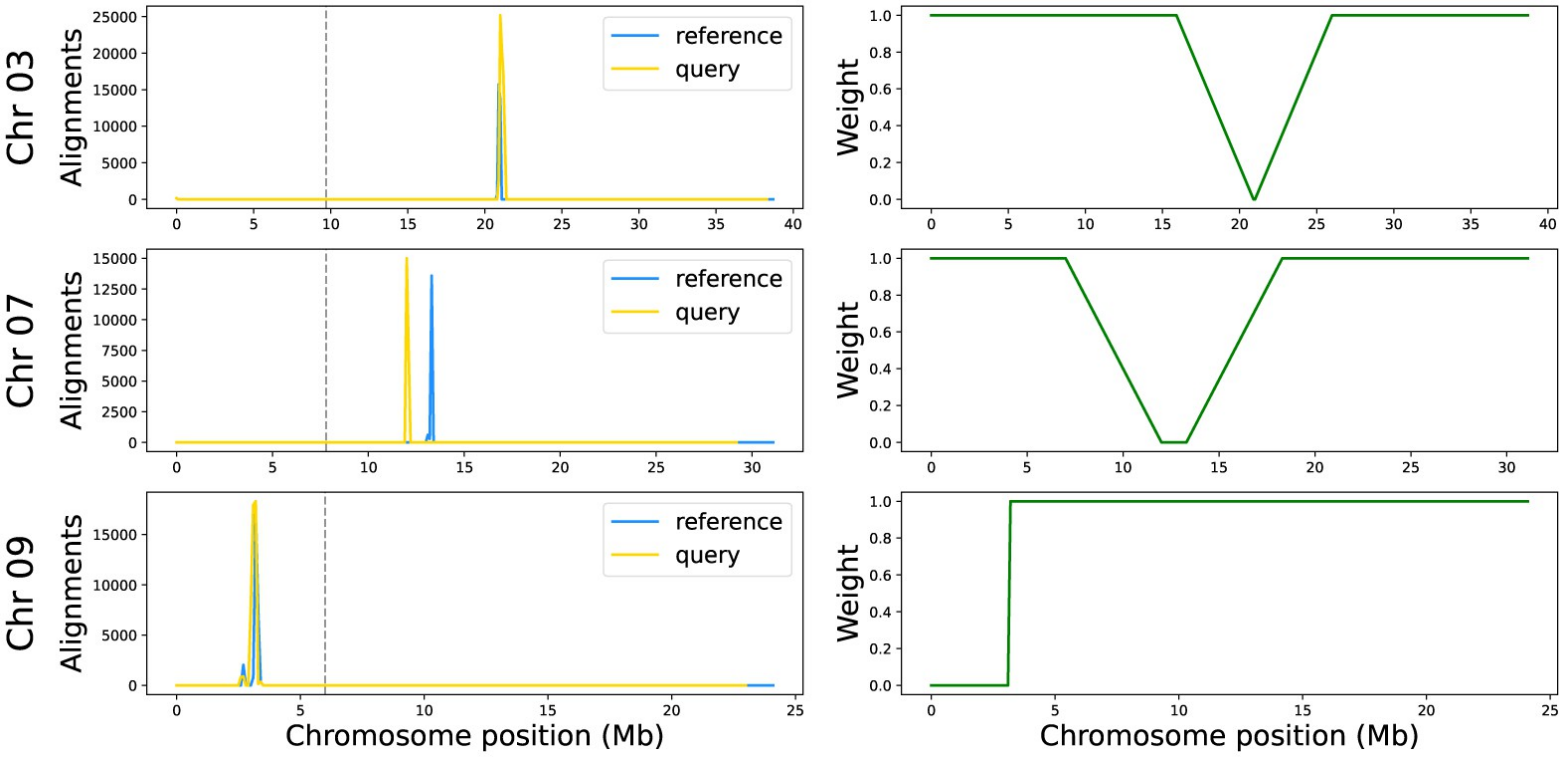
Centromere detection. Centromere detection using CentO sequences and CentO-based centromere correction distribution for rice chromosomes 3, 7 and 9. The plots on the left show the count of CentO alignments in 100 kb windows for the reference sequence in blue and the query sequence in yellow. The vertical gray dashed line indicates a quarter of the chromosome length, which is used to identify whether the chromosome is metacentric or telocentric and thus choose the weight function for centromere correction. The graphs on the right show the weight function in green applied for each case, at the top when the two peaks are together, in the middle when they are separated and at the bottom when these two peaks are before the quarter of the chromosome.

Next, a weight function is constructed to correct the predictive values near the boundaries of the centromere (see Fig 5), where recombination is expected to be lower than in the rest of the chromosome. This function maps to zero all the values between *c*_0_ and *c*_1_. The values of the 50 windows further to the left (right) of *c*_0_ (*c*_1_) are multiplied by a decreasing (increasing) linear function with minimum value zero and maximum value one. There is a special case of telomeric chromosomes having a Nucleolar Organizer Region (NOR) on the short arm, which is known to block recombination [26, 27]. In this case all values to the left of *c*_1_ are mapped to zero while values to the right are mapped to one. The latter should be considered only when the centromeric region is within the first quarter of the chromosome (e.g., rice chromosome 9). Therefore, two weight functions are defined, a function *f* for centromeric chromosomes, and a function *g* for the telomeric chromosomes. Both functions are defined from the set of windows *W*, with function *f* mapping to the closed real interval [0, 1], and function *g* mapping to the set {0, 1} as follows:

$$\begin{array}{c} f\left(w\right)=\{\begin{array}{ccc} 1 & , & 0\leq Id_{2}\left(w\right)\leq c_{0}-50\\ \frac{-1}{50}\left(w-c_{0}\right) & , & c_{0}-50<Id_{2}\left(w\right)\leq c_{0}\\ 0 & , & c_{0}<Id_{2}\left(w\right)\leq c_{1}\\ \frac{1}{50}\left(w-c_{0}\right) & , & c_{1}<Id_{2}\left(w\right)\leq c_{1}+50\\ 1 & , & c_{1}+50<Id_{2}\left(w\right)<n \end{array} \end{array}$$ (7)

$$\begin{array}{c} g\left(w\right)=\{\begin{array}{cc} 0 & 0\leq Id_{2}\left(w\right)<c_{1}\\ 1 & c_{1}\leq Id_{2}\left(w\right)\leq n \end{array} \end{array}$$ (8)

Finally, the identity values from *Id*_2_ are corrected by the function *Id*_3_: *W* → [0, 2], using the weight functions *f* and *g* as follows:

$$\begin{array}{c} Id_{3}\left(w\right)=\{\begin{array}{cc} Id_{2}\left(w\right)\cdot f\left(w\right) & c_{1}>n/4\\ Id_{2}\left(w\right)\cdot g\left(w\right) & \text{otherwise,} \end{array} \end{array}$$ (9)

where *c*_1_, as defined above, is the left boundary of the centromere, and *n* is the total number of windows of the reference chromosome.

#### Step 4: Smoothing

The fourth step, consisting of applying a special adaptation of exponential smoothing that replaces the value of the first window with zero, allows the prediction of the recombination rate to start at zero as actually occurs in the experimental data. Here *α* = 0.1 is used as defined in Section Testing hypothesis for the usual exponential smoothing. Thus, the final prediction of recombination is given by the function *Id*_4_, which maps the set of windows *W* to the real closed interval [0, 1] (i.e., *Id*_4_: *W* → [0, 1]). This function smoothes the identity values of *Id*_3_, for each window *w* ∈ *W*, as follows:

$$\begin{array}{c} Id_{4}\left(w\right)=\{\begin{array}{cc} 0 & w=0\\ \alpha Id_{3}\left(w\right)+\left(1-\alpha \right)Id_{4}\left(w-1\right) & w>0. \end{array} \end{array}$$ (10)

### Parameter optimization and model evaluation

The two metrics involved in the evaluation and calibration of the model are the Pearson correlation *r* and the coefficient of determination *R*^2^. Given data {(*x*_1_, *y*_1_), …, (*x*_*n*_, *y*_*n*_)} consisting of *n* pairs, these two metrics are defined as follows:

$$\begin{array}{c} r=\frac{\sum_{i=1}^{n}(x_{i}-\bar{x})(y_{i}-\bar{y})}{\sqrt{\sum_{i=1}^{n}(x_{i}-\bar{x}{)}^{2}\sum_{i=1}^{n}(y_{i}-\bar{y}{)}^{2}}} \end{array}$$ (11)

$$\begin{array}{c} R^{2}=1-\frac{\sum_{i=1}^{n}{\left(y_{i}-{\hat{y}}_{i}\right)}^{2}}{\sum_{i=1}^{n}{\left(y_{i}-\bar{y}\right)}^{2}} \end{array}$$ (12)

where $\bar{x}$ is the sample mean and $\hat{y}$ is the fitted linear regression between *x* and *y*.

The 7 model parameters (*p*_1_, *p*_2_, *p*_3_, *t*_1_, *t*_2_, *t*_3_, and *t*_4_) are adjusted by maximizing the coefficient of determination *R*^2^ between the final prediction of the model *Id*_4_ (see Eq 10) and the experimental recombination *X*_*s*_ (see Eq 2) of a single chromosome. The parameter optimization was done by the Sequential Least Squares Programming (SLSQP) minimizing (1 − *R*^2^). The model is adjusted from information on one chromosome and the adjusted model is used to predict recombination on the remaining 11 chromosomes. The prediction performance for each chromosome is evaluated based on the Pearson correlation *r*, and the coefficient of determination *R*^2^ between its output and the experimental recombination.

## Results and discussion

### Sequence identity versus recombination

**Fig 6:**
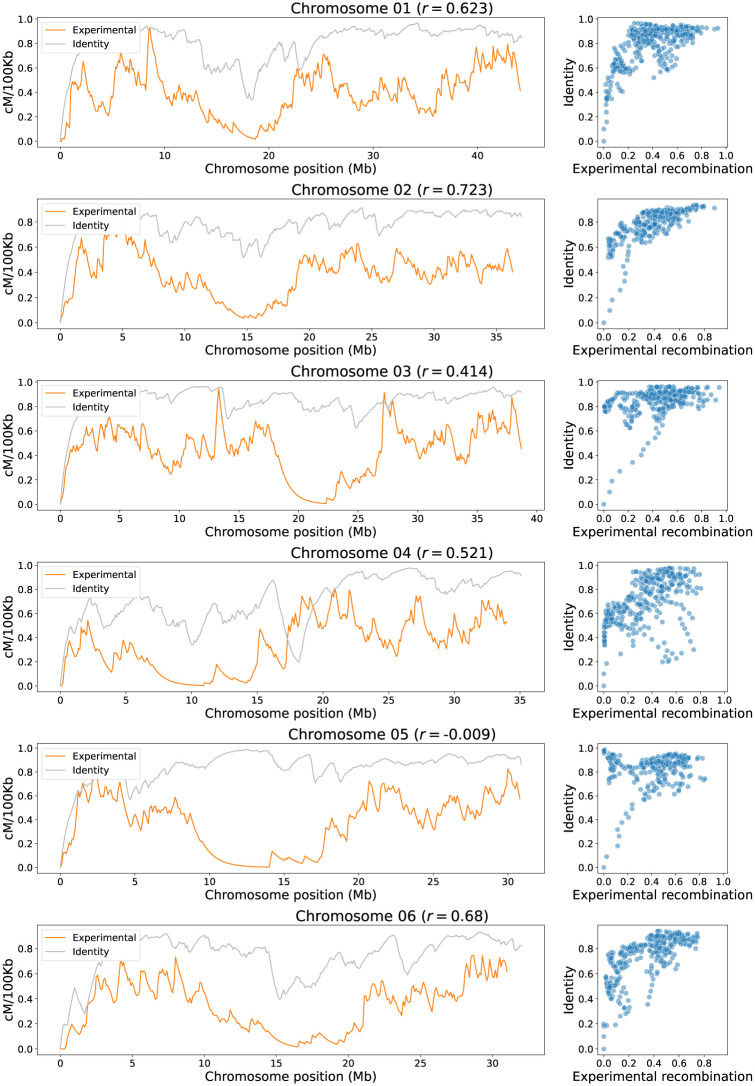
Identity correlation analysis for chromosomes 1 to 6 (cross IR64 x Azucena). On the left the landscape of experimental recombination (orange) and identity criteria (grey) are shown by windows of 100 kb along each chromosome. On the right scatterplots of experimental recombination vs. identity for each chromosome shows positive trends between them. The dots represent the 100 kb windows of the left graphs. The value of the corresponding Pearson correlation coefficient *r* is shown in parentheses next to the chromosome name.

**Fig 7:**
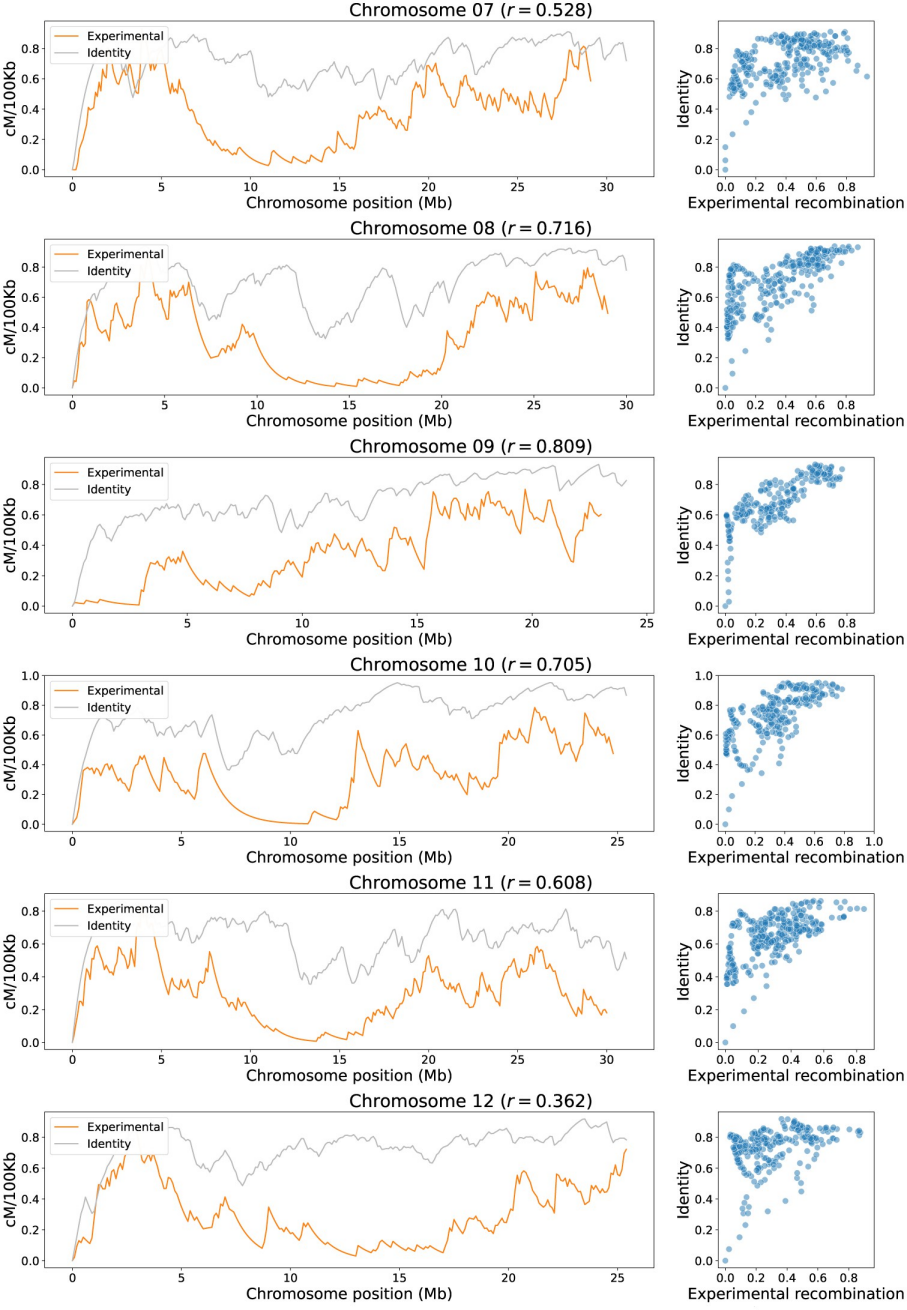
Identity correlation analysis for chromosomes 7 to 12 (cross IR64 x Azucena). On the left the landscape of experimental recombination (orange) and identity criteria (grey) are shown by windows of 100 kb along each chromosome. On the right scatterplots of experimental recombination vs. identity for each chromosome shows positive trends between them. The dots represent the 100 kb windows of the left graphs. The value of the corresponding Pearson correlation coefficient *r* is shown in parentheses next to the chromosome name.

The identity criteria values between parental chromosome sequences correlates positively with their progeny experimental recombination rates, as shown in Figs 6 and 7. These positive correlations are not complete because several windows move away from the linear relationship; however, it contains enough information to show trends. This supports the hypothesis that similar genome regions recombine more frequently than regions with higher structural difference [28, 29], a relationship that could explain several evolutionary mechanisms. The identity *sensus stricto* measures the ratio of identical bases between two sequences and can accurately represent the structural variability because every base that is not equal between sequences is marked as a variant, inversion, or absent base. This even eliminates a common problem such as repetitive sequences because they are absorbed by the identity measure. The identity is in great proportion conditioned by the alignment process. A good alignment process by itself is not sufficient for a proper identity estimation, because contigs do not follow a strict pattern due to structural rearrangements. As a consequence, the resulting alignment is filled with paired and unpaired regions, and in many cases with inversion events or overlapping, without counting on the abundant variants such as SNPs and indels polymorphisms. Therefore, a protocol, which allows to quantify the identity and other variables using a windows-based approach, is developed.

The mean correlation between recombination rates and sequence identity evaluated for the 12 rice chromosomes in the IR64 x Azucena cross is *r* = 0.56±0.21. This positive correlation is important because a single variable is supporting a considerable magnitude of the explanation. However, identity is an aggregated variable that implicitly carries the information of other structural variables. More specifically, identity is the ratio of bases that do not correspond to variants, inversions, or absent bases within a genome interval.

The higher correlations are found on chromosomes 9 and 2 with 0.809 and 0.723 respectively; meanwhile, lower correlations are found on chromosomes 5 and 12 with −0.009 and 0.362, respectively, being Chromosome 5 the unique with near zero, negative correlation. This can be explained because the alignment of Chromosome 5 between these two varieties has a high identity in the centromere region, originating a trend opposite to that observed in other chromosomes, which usually report low identity values in centromeric regions.

In Figs 6 and 7 can also be noted, for each scatterplot, a set of points with low identity values that align almost in a straight line with the experimental recombination values. In theory, these points may have an effect by increasing the correlation scores between the identity and experimental recombination. However, to rule this situation out, points with identity values less than 0.4 were removed and the correlation recalculated. It was found that the correlations increased in all chromosomes, except in Chromosome 9, where the correlation decreased by 0.059. Chromosome 1 had the smallest increase in correlation with a gain of 0.045, while Chromosome 5 had the highest increase in correlation with a 0.708 gain. This indicates that the inclusion of these data with low identity in the analysis does not increase the correlation values, which gives reliability to the analysis performed with all the data.

Sequence identity by itself can reproduce some peaks and valleys of the recombination landscape, indicating that recombination is greatest in regions where identity between genomes is greatest and least where it is not. Thus, if genomic identity is highly correlated with chromosomal recombination, it can be used as a starting point for the construction of a model whose aim is to predict recombination. In consequence, a model based on sequence identity was developed.

### Parameter optimization and model evaluation

The model was calibrated on each of the twelve chromosomes. Each calibration resulted in a different set of optimal parameters shown in Table 1.

**Table 1:** Parameters for each model calibration.

parameter	chr01	chr02	chr03	chr04	chr05	chr06	chr07	chr08	chr09	chr10	chr11	chr12
*p*_1_	0.529	0.480	0.568	0.563	0.470	0.469	0.508	0.453	0.476	0.467	0.380	0.504
*p*_2_	1.000	0.000	0.102	1.000	0.000	0.998	1.000	0.000	0.000	0.000	0.000	1.000
*p*_3_	1.000	1.000	0.500	0.666	1.000	1.000	0.700	0.100	0.500	1.000	1.000	0.537
*t*_1_	0.970	0.960	0.950	0.940	0.940	0.970	0.930	0.940	0.920	0.960	0.920	0.940
*t*_2_	0.900	0.300	1.000	0.100	0.600	0.900	0.600	1.000	0.700	0.300	0.700	0.900
*t*_3_	0.000	0.000	0.100	0.000	0.000	0.000	0.100	0.100	0.100	0.000	0.000	0.000
*t*_4_	0.002	0.002	0.001	0.004	0.002	0.002	0.005	0.004	0.001	0.002	0.005	0.003

The columns indicate the chromosome on which the model was calibrated and its corresponding set of optimum parameters.

**Fig 8:**
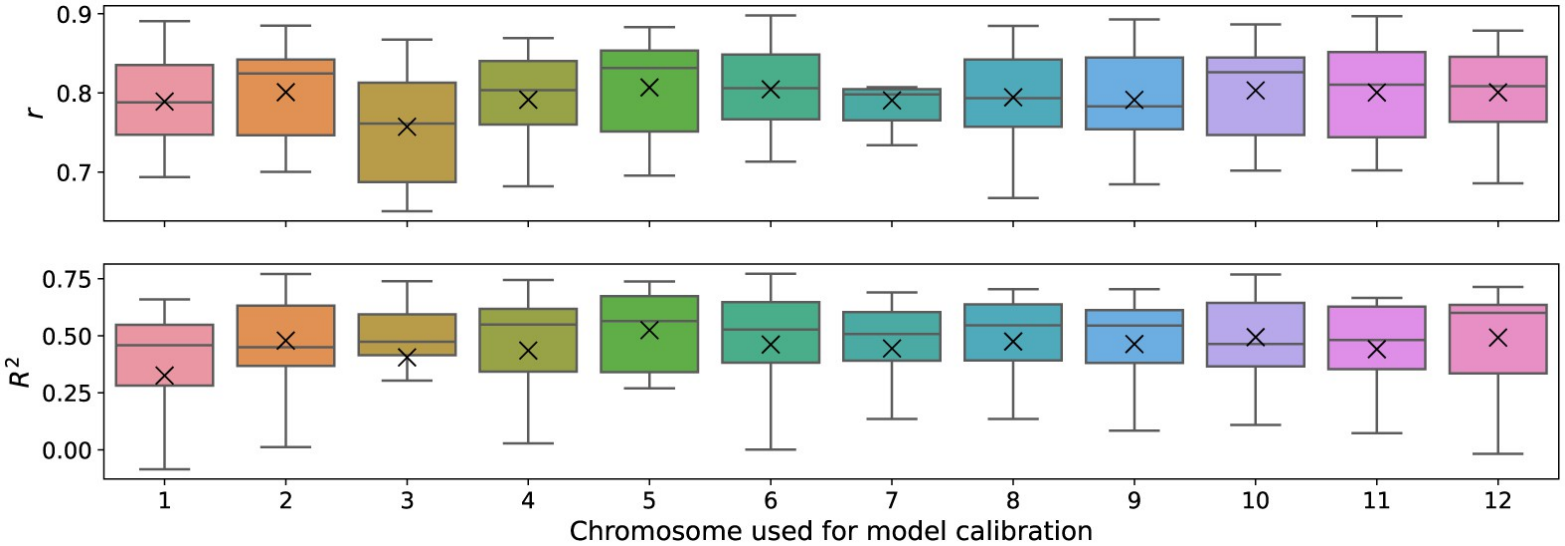
Boxplot distributions of model performance. Distributions of correlation *r* and coefficient of determination *R*^2^ show that there is no significant difference in recombination predictions when the model is calibrated on different chromosomes. Each boxplot represents the values obtained for the remaining 11 chromosomes when the model was calibrated on the indicated chromosome.

The 12 model calibrations were used to test the prediction on the remaining eleven chromosomes. Fig 8 shows the distribution of the values *r* and *R*^2^ obtained when evaluating the twelve predictions of each model calibration. The results look similar in all cases for both *r* and *R*^2^. Furthermore, a two-sample Kolmogorov-Smirnov test, was performed between the evaluations of each pair of model calibrations. The test output indicated that the difference between the *R*^2^ distributions is not statistically significant (all *p*-values > 0.05). The same happens with the distributions of *r* (all p-values >0.05). Therefore, the 12 distributions of *R*^2^ are not significantly different from each other, nor are the 12 distributions of *r*. This means that using the model calibrated on any arbitrarily chosen chromosome does not generate significant changes in the prediction performance.

### Predictions

For practical reasons, some results discussed below are focused on the prediction obtained with the model calibrated on Chromosome 1, which turns out to be the longest chromosome and therefore the one that provides the greatest amount of data for calibration. Nevertheless, recall that all 12 calibrations have been used and consistent results have been obtained.

Overall, for all 12 calibrations of the model, the predicted recombination have a correlation of *r* = 0.8 ± 0.06 and a coefficient of determination *R*^2^ = 0.45 ± 0.25, which shows the power of the model to reproduce recombination trends along chromosomes. In terms of correlation, the lowest average value belongs to the model calibrated with chromosome 3 (*r* = 0.757 ± 0.074). The lowest average coefficient of determination belongs to the model calibrated with Chromosome 1 (*R*^2^ = 0.326 ± 0.408, *r* = 0.789 ± 0.065). While, the model calibrated with Chromosome 5 has the highest average performance for both evaluation metrics: *r* = 0.807 ± 0.065 and *R*^2^ = 0.524 ± 0.17. It should be noted that the correlation on the calibrated chromosome (*r* = 0.708) is lower than the correlations of the remaining predictions on the other 11 chromosomes (*r* = 0.796 ± 0.063). The latter indicates that this model is not overfitted to the observed data and is capable of predicting recombination rates of independent datasets, even achieving better performance.

**Fig 9:**
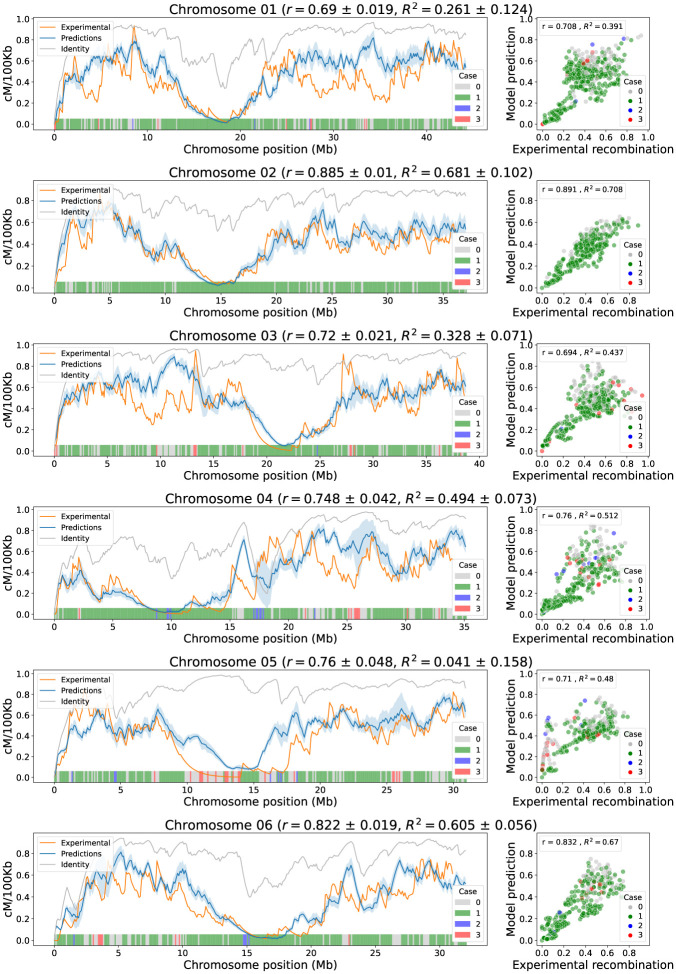
Model correlation analysis in chromosomes 1 to 6 (cross IR64 x Azucena). On the left, the landscape of experimental recombination (orange), identity criteria (grey), and predicted recombination calibrated with chromosome 1 (blue) are shown by windows of 100 kb along each chromosome. The shaded blue band represents the standard deviation of the predictions for different calibrations, and the mean correlations and coefficients of determination are presented next to the chromosome name. The colored bars at the bottom indicate which case from Step 1 of the model is applied to each window. On the right, for each chromosome, a scatterplot of experimental vs. predicted recombination calibrated with Chromosome 1 shows positive trends between them. The dots represent the 100 kb windows of the graphs on the left and colors indicate the case that was applied in the first step of the model for each window. Inside each boxplot, the correlation and coefficient of determination values between model prediction and experimental recombination using the calibration in Chromosome 1 is presented.

**Fig 10:**
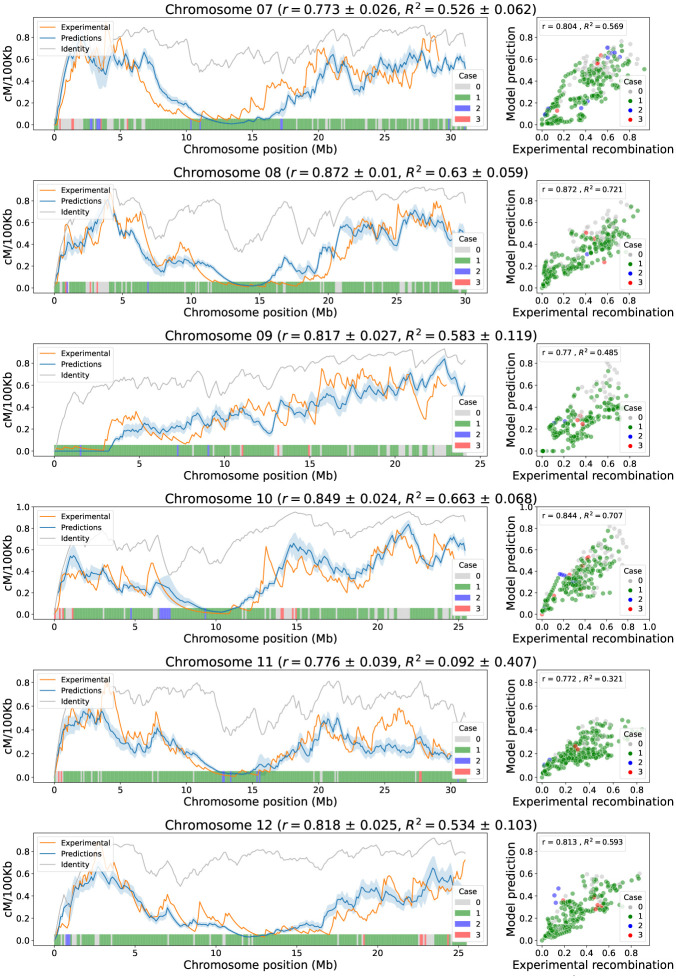
Model correlation analysis in chromosomes 7 to 12 (cross IR64 x Azucena). On the left, the landscape of experimental recombination (orange), identity criteria (grey), and predicted recombination calibrated with Chromosome 1 (blue) are shown by windows of 100 kb along each chromosome. The shaded blue band represents the standard deviation of the predictions for different calibrations, and the mean correlations and coefficients of determination are presented next to the chromosome name. The colored bars at the bottom indicate which case from the first step of the model is applied to each window. On the right, for each chromosome, a scatterplot of experimental vs. predicted recombination calibrated with Chromosome 1 shows positive trends between them. The dots represent the 100 kb windows of the graphs on the left and colors indicate the case that was applied in the first step of the model for each window. Inside each boxplot, the correlation and coefficient of determination values between model prediction and experimental recombination using the calibration in Chromosome 1 is presented.

Figs 9 and 10 depict, on the left, the landscape for the experimental recombination, identity, and model predictions. The shaded blue band on each chromosome represents the standard deviation of the predictions made with the 12 calibrated models. The width of these bands indicates that the predictions from any of the model calibrations are consistent across all chromosomes. Figs 9 and 10 depict, on the right, the linear relationship between the experimental recombination and the prediction of the model calibrated with Chromosome 1. These linear relationships between the model predictions and the experimental recombination are greater than those obtained with the identity, showing less dispersion in the scatter plot and higher correlation coefficients, and indicating that the model outputs can better reproduce the data trends. The marker color in the scatter plot, and the bar color at the bottom of the line plots, represents the case of the model that was applied in a specific window.

It is important to analyze the incidence of the cases, from Step 1 of the model, in the prediction of recombination. For all chromosomes, regardless of model calibration, the first case is the most applied in 68.5% of the chromosome windows on average, followed by the non-application of any case 25.8%. Meanwhile, the cases two and three are the least applied, with an average of 3.5% and 2.1%, respectively. This indicates that the first case of Step 1 is the one that contributes the most to the prediction of the model for all chromosomes, allowing the formation of medium and low recombination regions. Despite the fact that cases two and three have a low incidence in the chromosomal windows, they help to define particular areas that escape the action of the first case.

**Fig 11:**
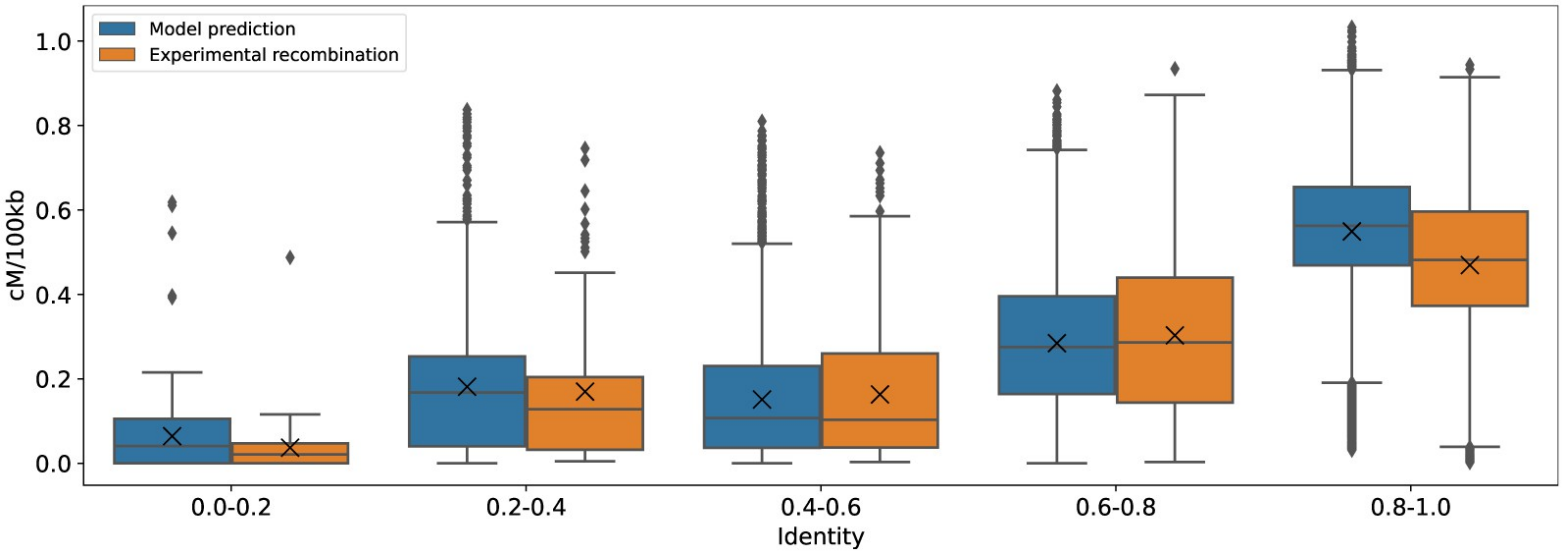
Distributions of experimental recombination and model predictions. Distributions of experimental and predicted recombination values according to the value of the window identity criteria. The boxes show that the values between them are similar at different magnitudes of identity.

**Fig 12:**
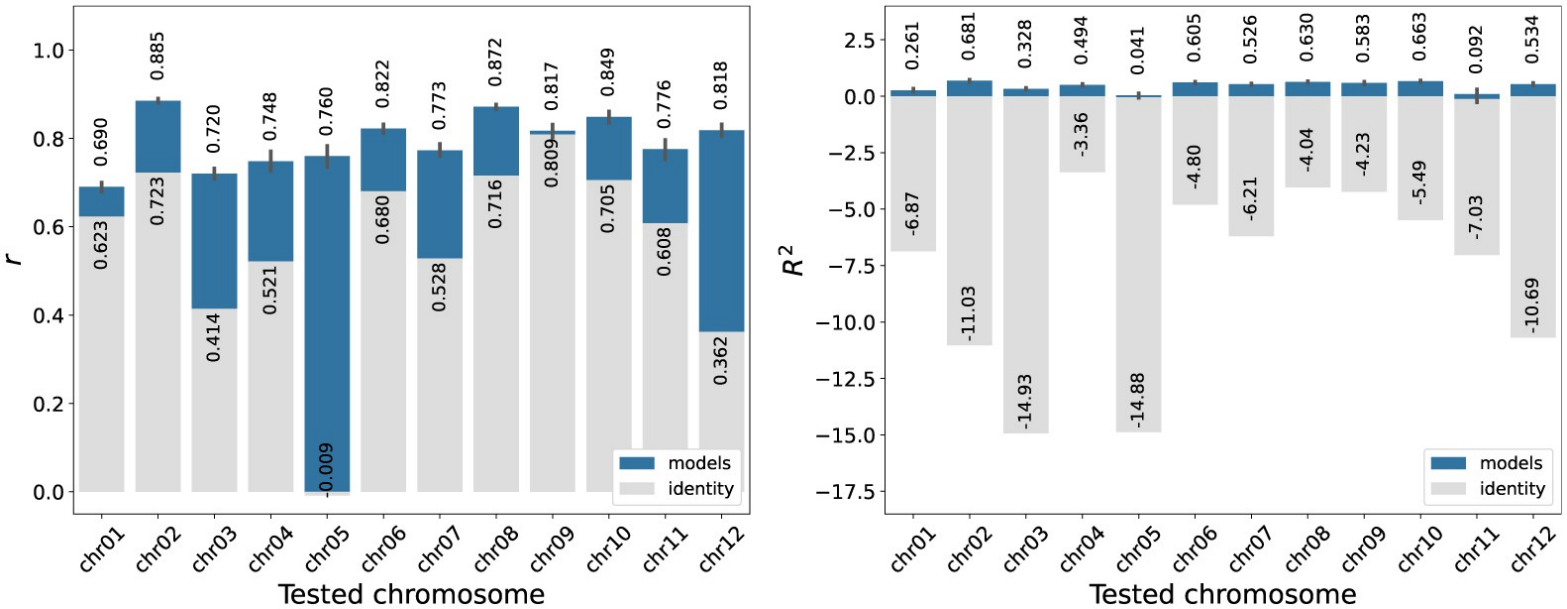
Gains in model performance versus identity. Correlation *r* (left) and coefficient of determination *R*^2^ (right) of identity criteria and model predictions with respect to recombination rates from 12 rice chromosomes (IR64 x Azucena cross). Base value of identity in gray, the gain of the model in blue. The graphs show the gain in recombination prediction for each chromosome when the model is used.

Both experimental recombination and predictions are similarly distributed according to the identity in Fig 11. Note that, with respect to identity, the proposed model markedly increased the correlation and the coefficient of determination, as shown in Fig 12. The average increase in correlation, across all calibrations and tested chromosomes, is 0.237 ± 0.197, meanwhile the increase in the coefficient of determination is 8.25±3.84, being the gain of prediction different for each chromosome. This gain is obtained because the different steps of the model transform the identity values of each 100 kb window, which helps to better represent peaks and valleys in the chromosomal arms and, in general, to identify the centromeric regions.

Chromosome 5 is an extreme case gaining 0.769 ± 0.048 correlation points with respect to identity. Other chromosomes with a high gain in correlation are 12 and 3, gaining 0.457 ± 0.025 and 0.306 ± 0.021 correlation points, respectively. These chromosomes, unlike the remaining 9 chromosomes, do not show a decreasing trend of identity near the centromere region. However, the application of the cases in Step 1 (see color bars in Figs 9 and 10), together with centromere correction, best approximate experimental recombination.

**Fig 13:**
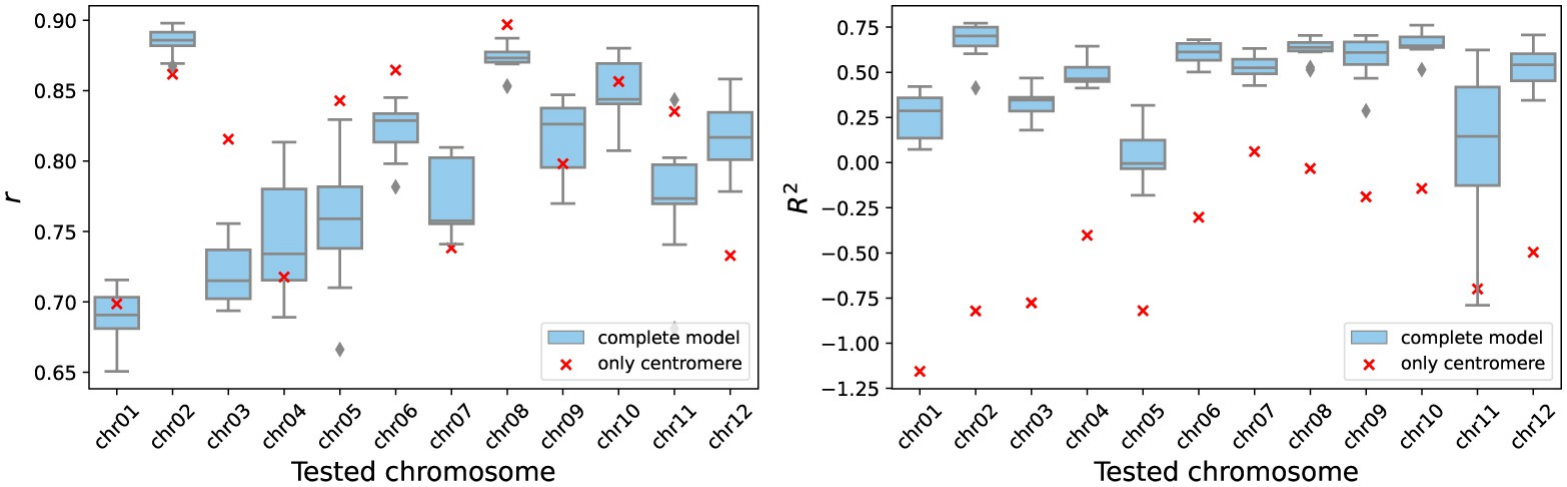
Performance with the complete model and only centromere correction. The graph on the right shows the correlation values between the recombination prediction and its experimental value when the full model is used and when only the centromere correction is used. Meanwhile, the left graph shows the coefficients of determination for the same comparisons. Although the correlations show a similar trend between the two experiments, the prediction is not satisfactory because the determination coefficients are all negative.

It may seem that centromere divergence has a great influence on the model prediction, since chromosomes with high centromere identity values have higher correlation gains. However, applying only the centromere correction to the identity does not produce satisfactory results (see Fig 13). Although this best approximates the trend of experimental recombination on chromosomes 3, 5, 6, 8, and 11 having a higher correlation, it fails to predict recombination values on all chromosomes. More specifically, when applying only the centromere correction the coefficient of determination *R*^2^ is in average −0.482 ± 0.376 for all chromosomes, whereas when applying the complete model the average *R*^2^ is 0.453 ± 0.255 for all calibrations applied to all chromosomes.

Chromosome 9 presents the extreme case of the lowest gain in correlation. This corresponds to a gain of only 0.008 ± 0.027 correlation points across all model calibrations. This means that the sequence identity is sufficient for Chromosome 9 to describe recombination rates, even approaching the mean correlation achieved by the model.

Chromosome 9 is unique with its telomeric centromere in rice and is treated differently in the third step of the model, avoiding the centromere correction applied to the other chromosomes. This special treatment is due to the existence of the Nucleolar Organizer Region (NOR) in the short arm of the chromosome. The NOR of Chromosome 9 is widely known to be a region where recombination is suppressed in rice [26], hence the special centromere correction. However, the effect of this correction in the Chromosome 9 prediction is focused on the short arm only, and the prediction on the long arm is completely determined by the other steps of the model. Although sequence identity by itself can generate a high correlation with the recombination rate for this cross (IR64 x Azucena) on Chromosome 9, the predictive values of the model continue to be preferred since the magnitude of the values is closer to those of recombination Fig 10.

Finally, it should be noted that the model predictions reach a high correlation rate for all the chromosomes evaluated; the model is able to reproduce the recombination landscape of the rice varieties IR64 and Azucena crossing.

## Conclusion

The results presented in this paper show that the proposed criteria for sequence identity is strongly correlated with chromosomal recombination. The strength of this correlation supports the introduction of a model based on window “identities”, which is shown to accurately predict recombination rates along the length of chromosomes. The model is developed using data on the first chromosome of rice (accessions IR64 and Azucena). It is cross-validated using the remaining eleven chromosomes. Across all 12 chromosomes, an average correlation of about 80% between experimental and prediction rates is achieved. Similar results are found when model training is performed on other chromosomes, being of great importance the gain in the determination coefficient.

The goal of this model is to enable the prediction of chromosome recombination landscapes among rice varieties using only the parental genomes as a source. Such an approach is particularly useful for breeding purposes, for it offers the potential to optimize crossing experiments. In particular, model prediction could allow to identify varieties that should better recombine than others with recipient genomes and to uncover recombination hot spots of vertical gene transfer. Predictions between rice varieties using this model should give good results because the model was developed using information from two genetically distant varieties, which is an extreme case compared to traditional crosses normally made in related lines.

The ultimate goal of the proposed model is to help breeders to reduce costs and execution times of crossing experiments. It is to explore, as a future project, the open path to use the model on other rice varieties, cereal species, and even on the broader spectrum of plants and animals.

## Supporting information

S1 File Experimental recombination. Experimental recombination values for the 12 rice chromosomes, Azucena x IR64 cross, in 100 kb windows.
(CSV)
